# Supplementary material for: Time-series transcriptome analysis identified differentially expressed genes in broiler chicken infected with mixed Eimeria species
Source: Front Genet. 2022 Aug 8;13:886781. doi: 10.3389/fgene.2022.886781 (PMC9393255; doi:10.3389/fgene.2022.886781)
Supplement: Supplementary file 2 [file DataSheet1.ZIP › 4dpi_KEGG.Gsea.1625100298584/KEGG_PPAR_SIGNALING_PATHWAY.html]

Details for gene set KEGG\_PPAR\_SIGNALING\_PATHWAY[GSEA]

|  || Dataset | TMM\_4dpi\_gct\_format\_4dpi\_gct\_format.Class\_4dpi.cls #PC\_versus\_NC.Class\_4dpi.cls #PC\_versus\_NC\_repos |
| Phenotype | Class\_4dpi.cls#PC\_versus\_NC\_repos |
| Upregulated in class | 1 |
| GeneSet | KEGG\_PPAR\_SIGNALING\_PATHWAY |
| Enrichment Score (ES) | 0.4713305 |
| Normalized Enrichment Score (NES) | 1.6414455 |
| Nominal p-value | 0.0070635723 |
| FDR q-value | 0.18255126 |
| FWER p-Value | 0.6538 |
Table: GSEA Results Summary

  

Fig 1: Enrichment plot: KEGG\_PPAR\_SIGNALING\_PATHWAY      
 Profile of the Running ES Score & Positions of GeneSet Members on the Rank Ordered List

  

| SYMBOL | TITLE | RANK IN GENE LIST | RANK METRIC SCORE | RUNNING ES | CORE ENRICHMENT || 1 | FABP6 | na | 129 | 1.521 | 0.0435 | Yes |
| 2 | ACSL5 | na | 234 | 1.287 | 0.0808 | Yes |
| 3 | GK2 | na | 398 | 1.062 | 0.1051 | Yes |
| 4 | PCK1 | na | 446 | 1.020 | 0.1376 | Yes |
| 5 | FABP5 | na | 504 | 0.960 | 0.1671 | Yes |
| 6 | APOA1 | na | 589 | 0.889 | 0.1918 | Yes |
| 7 | FABP2 | na | 653 | 0.848 | 0.2168 | Yes |
| 8 | CPT1A | na | 785 | 0.765 | 0.2332 | Yes |
| 9 | ACSL4 | na | 863 | 0.731 | 0.2528 | Yes |
| 10 | LPL | na | 954 | 0.691 | 0.2700 | Yes |
| 11 | SCD | na | 1084 | 0.644 | 0.2822 | Yes |
| 12 | FABP3 | na | 1105 | 0.636 | 0.3032 | Yes |
| 13 | ACSL1 | na | 1172 | 0.612 | 0.3196 | Yes |
| 14 | SCP2 | na | 1208 | 0.601 | 0.3381 | Yes |
| 15 | PPARA | na | 1223 | 0.596 | 0.3582 | Yes |
| 16 | ANGPTL4 | na | 1324 | 0.568 | 0.3701 | Yes |
| 17 | CD36 | na | 1326 | 0.568 | 0.3903 | Yes |
| 18 | PLIN1 | na | 1330 | 0.567 | 0.4103 | Yes |
| 19 | ACSL3 | na | 1396 | 0.548 | 0.4244 | Yes |
| 20 | SORBS1 | na | 1415 | 0.543 | 0.4423 | Yes |
| 21 | ACSL6 | na | 1433 | 0.536 | 0.4600 | Yes |
| 22 | FABP4 | na | 1519 | 0.515 | 0.4713 | Yes |
| 23 | CPT2 | na | 1915 | 0.437 | 0.4539 | No |
| 24 | RXRA | na | 2286 | 0.377 | 0.4364 | No |
| 25 | SLC27A6 | na | 2379 | 0.365 | 0.4418 | No |
| 26 | FABP1 | na | 2389 | 0.363 | 0.4540 | No |
| 27 | ADIPOQ | na | 2680 | 0.326 | 0.4414 | No |
| 28 | FADS2 | na | 2762 | 0.313 | 0.4458 | No |
| 29 | NR1H3 | na | 3213 | 0.255 | 0.4173 | No |
| 30 | ACOX3 | na | 3827 | 0.184 | 0.3726 | No |
| 31 | ILK | na | 4712 | 0.105 | 0.3024 | No |
| 32 | RXRG | na | 4744 | 0.102 | 0.3035 | No |
| 33 | ACOX1 | na | 4782 | 0.099 | 0.3039 | No |
| 34 | PDPK1 | na | 5471 | 0.039 | 0.2478 | No |
| 35 | PPARG | na | 7261 | -0.114 | 0.1023 | No |
| 36 | DBI | na | 7319 | -0.120 | 0.1018 | No |
| 37 | ACOX2 | na | 7988 | -0.178 | 0.0523 | No |
| 38 | ACAA1 | na | 8300 | -0.208 | 0.0337 | No |
| 39 | SLC27A2 | na | 8317 | -0.210 | 0.0399 | No |
| 40 | PLTP | na | 8531 | -0.233 | 0.0304 | No |
| 41 | CYP4A22 | na | 8776 | -0.259 | 0.0192 | No |
| 42 | SLC27A4 | na | 9160 | -0.304 | -0.0020 | No |
| 43 | CYP8B1 | na | 9820 | -0.389 | -0.0432 | No |
| 44 | PCK2 | na | 10229 | -0.450 | -0.0612 | No |
| 45 | EHHADH | na | 10699 | -0.538 | -0.0812 | No |
| 46 | ACADL | na | 11004 | -0.609 | -0.0849 | No |
| 47 | PPARD | na | 11032 | -0.617 | -0.0651 | No |
| 48 | ME1 | na | 11084 | -0.631 | -0.0469 | No |
| 49 | SCD5 | na | 11309 | -0.707 | -0.0404 | No |
| 50 | CYP27A1 | na | 11384 | -0.740 | -0.0201 | No |
| 51 | SLC27A1 | na | 11586 | -0.861 | -0.0062 | No |
| 52 | FABP7 | na | 11837 | -1.164 | 0.0145 | No |
Table: GSEA details [plain text format]

  

Fig 2: KEGG\_PPAR\_SIGNALING\_PATHWAY      
 Blue-Pink O' Gram in the Space of the Analyzed GeneSet

  

Fig 3: KEGG\_PPAR\_SIGNALING\_PATHWAY: Random ES distribution      
 Gene set null distribution of ES for **KEGG\_PPAR\_SIGNALING\_PATHWAY**
